# Unlocking personalized endometrial cancer treatment: the critical role of the BBIRE biobank in sample collection and distribution

**DOI:** 10.3389/fmolb.2026.1748347

**Published:** 2026-02-12

**Authors:** V. Bruno, M. Betti, L. Ciuffreda, A. M. B. Arteni, M. Ferretti, F. Rossi, C. Accetta, C. Mandoj, V. Laquintana, F. De Nicola, S. Donzelli, S. Vaccarella, A. Di Maio, T. Mancuso, M. Haoui, M. Carosi, G. Cigliana, E. Pescarmona, M. Fanciulli, G. Piaggio, E. Vizza, M. Pallocca, G. Ciliberto, G. Blandino, S. Di Martino

**Affiliations:** 1 Gynecologic Oncology Unit, Department of Experimental Clinical Oncology, IRCCS Regina Elena National Cancer Institute, Rome, Italy; 2 Biostatistics, Bioinformatics and Clinical Trial Center, IRCCS Regina Elena National Cancer Institute, Rome, Italy; 3 Gene Expression and Cancer Model Unit, Department of Research, Advanced Diagnostics and Technological Innovation, Translational Research Area, IRCCS Regina Elena National Cancer Institute, Rome, Italy; 4 Department of Pathology Unit, Tissue Biobank, IRCCS Regina Elena National Cancer Institute, Rome, Italy; 5 Clinical Pathology Unit and Cancer Biobank, IRCCS Regina Elena National Cancer Institute, Rome, Italy; 6 Translational Oncology Research Unit, IRCCS Regina Elena National Cancer Institute, Rome, Italy; 7 Instituto degli Endotipi in Oncologia, Metabolismo e Immunologia “G. Salvatore” (IEOMI), National Research Council, Naples, Italy; 8 IRCCS Regina Elena National Cancer Institute, Rome, Italy

**Keywords:** biobanking, digital pathology, endometrial cancer, multiomics, patient derived organoids, precision oncology, translational oncology

## Abstract

**Introduction:**

Endometrial cancer (EC) is the most common gynecological malignancy and the sixth most common cancer in women. Although it primarily affects women around or after menopause, an increasing number of cases are now being found in women of reproductive age. This shift highlights the need for fertility-sparing treatments and research.

**Methods:**

The tumor biobank of the Regina Elena National Cancer Institute (BBIRE) has played a central role in EC research by simplifying the collection and distribution of high-quality samples linked to clinical data. BBIRE follows strict protocols and uses secure databases to protect patient privacy, meet regulations, and keep clinical information accurate. These steps help maintain sample quality and reduce errors before analysis.

**Results:**

This research highlights the importance of the BBIRE-tissue processing group in the coordinated management of 545 gynecological tumor samples, comprising 321 EC samples, underscoring its importance as a crucial instrument for translational research. The biobank supports a complete research process, from patient enrollment to molecular data analysis. Its flexible, standardized structure helps ensure reliable results in different research settings.

**Discussion:**

As a gynecologic oncology resource, BBIRE facilitates large-scale studies and collaboration among team researchers. This support is essential for identifying new biomarkers, tailoring treatments, and advancing precision medicine. The development of personalized care and improved outcomes for women with EC can be accelerated when work is performed collaboratively by surgeons, biobanks, and researchers.

**Statement of Significance:**

The BBIRE Biobank is a game changer in cancer research that enables the integration of annotated samples, multiomics data, and organoid models to identify molecular drivers and accelerate personalized care.

## 1 Introduction

In the past decade, technological advances and new methodologies have transformed oncology research. Improving cancer research and treatment requires high quality samples and reliable data management. As a result, both basic and translational research now depend on the collection, processing, storage, tracking, and distribution of samples (Müller et al., 2020). Strategic management of samples is essential because emerging technologies, such as high throughput molecular profiling and artificial intelligence (AI), produce large and complex datasets. In this context, establishing integrated biobank networks has become a critical strategy for supporting preclinical and clinical research (Blow, 2009; Best Practices for Biospecimen Resources - NCI, 2025).

Since its establishment in 2014, BBIRE has ensured the safety, privacy, and traceability of samples through standard operating procedures (SOPs), barcode tracking, and encrypted databases. These datasets include clinicopathologic, epidemiologic, demographic, and multiomics data, all of which are essential for comprehensive cancer research (Bonizzi et al., 2022; Braun et al., 2014; Leff and Yang, 2015; Luo et al., 2014; Saifuddin et al., 2017). With the advancement of AI, data can now be improved more efficiently through automatic annotation and quality control, which results in more accurate, patient centered healthcare and quicker advancements in customized cancer treatments (Frascarelli et al., 2023). The quality of the samples, particularly their cellular composition, is essential for their effective use in research. At BBIRE, we ensure sample integrity and adequate representation of target cells by implementing rigorous quality control measures before and after storage and molecular analyses. To this end, digital pathology is employed to evaluate sample composition and quality and minimize observer variability via AI-driven image analysis. This approach enhances the accuracy of histopathological evaluations and sample selection, promotes reproducibility, and facilitates personalized treatment decisions (Wei and Simpson, 2014; Gallo et al., 2024). The use of patient derived organoids (PDOs) adds value to the biobank. In fact, organoid technology promotes the development of integrated biobank networks and enhances the translational potential of biobank samples, which accelerates cancer research and streamlines research workflows.

EC has emerged as a significant public health problem, particularly in women under 40 years of age, with increasing diagnosis rates and a notable association with nulliparity (Abdol Manap et al., 2022; National Cancer Institute, 2023). In 2024, approximately 8,652 new cases of EC were diagnosed in Italy, representing approximately 4.9% of all cancer diagnoses in the country. According to AIOM data for 2024, among women, uterine cancer caused 3,720 deaths (6.8% of female cancer deaths), and ovarian cancer accounted for 3,472 deaths (6.3%) between 2006 and 2021 (Associazione Italiana di Oncologia Medica AIOM, 2024) (https://www.aiom.it). As the incidence of cancer continues to rise, understanding its molecular and genetic basis is crucial for the development of more targeted and effective therapies. This study highlights the critical role of the BBIRE Biobank in collecting and distributing samples from 545 female genital cancer patients, including 346 EC patients, to support translational cancer research, facilitate biomarker discovery, and advance the development of personalized diagnostics and therapies. The primary outcome of this study is to demonstrate the operational validation of the BBIRE biobank as a robust infrastructure for generating high-quality samples to support translational and precision oncology research in endometrial cancer. Specifically, the BBIRE biobank represents a distinctive resource that can be used to study tumor heterogeneity, hormone-related mechanisms, and treatment resistance in gynecologic malignancies, thereby supporting the development of more effective and personalized therapeutic approaches in EC.

## 2 Methods

### 2.1 BBIRE cancer tissue bank

**FIGURE 1:**
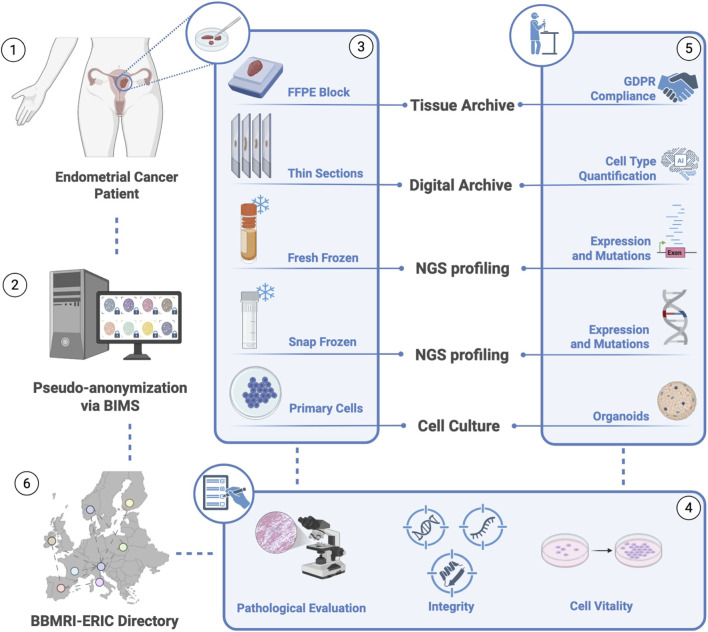
Overview of the endometrial cancer samples workflow. Tumor samples are collected and pseudo-anonymized (1–2), processed into multiple formats (FFPE, frozen tissues, thin sections, and primary cells (3), and subjected to quality control (4). EC samples are archived and used for downstream analyses, including NGS-based expression and mutation profiling, cell type quantification, and organoid generation, in compliance with GDPR (5). Sample metadata are registered in the BBMRI-ERIC directory (6).

BBIRE plays a crucial role in cancer research, systematically collecting and storing high quality samples. It was developed in collaboration with the Departments of Clinical Pathology and Pathology Unit, with the support of the Scientific Directorate. The BBIRE standardized approach ensures sample quality, consistency, and traceability. In collaboration with the gynecological surgical team, BBIRE collects EC tumor samples (tumor tissue, matching normal tissue, whole blood, plasma, serum and PBMCs) in accordance with the SOPs. All biological samples are processed and cryopreserved according to strict quality protocols to ensure their suitability for research within the BBIRE Biobank, which is accredited by the International Organization for Standardization (ISO). These operational processes comply with ISO 9001:2015 and CEN/TS quality standards. ISO 9001:2015 is an internationally recognized standard that provides a framework for designing, implementing, maintaining and improving quality management systems (QMSs). This standard emphasizes the importance of documented procedures, effective document and record control, internal audits, and corrective and preventive actions while focusing on an organization’s ability to meet customer requirements and increase customer satisfaction. Sample integrity is ensured through 2D barcode labeling, semiautomated cryostorage platforms and standardized, high-quality cryopreservation and processing systems. An independent scientific and ethical advisory board oversees the biobank’s operations, conducting regular audits to ensure compliance with ethical and quality assurance standards. Access is granted to approved projects that promote the use of diverse, well annotated samples. This centralized model improves sample availability, expands access for researchers, and increases the efficiency and quality of sample processing. The BBIRE framework directly supports the Institute’s mission to advance personalized cancer care through high quality research (Figure 1).

### 2.2 Patient identification, consent, and confidentiality

Patients who were diagnosed with EC and scheduled for surgical treatment (either conization or hysterectomy) according to the International Federation of Gynecology and Obstetrics (FIGO) staging criteria were prospectively recruited for this study (Berek et al., 2023). The study’s inclusion criteria were as follows: histological confirmation, a minimum age of 18 years, and the ability to provide informed consent. Before sample collection, all participants were thoroughly informed of the objectives of the study, including the collection of tissue and blood samples and associated clinical data for use in future translational research. To date, the BBIRE tissue biobank has played a central role in the collection and distribution of biological samples from various cases of female genital tract cancers, including EC. In accordance with the Declaration of Helsinki, written informed consent was obtained from each patient. The study protocol was approved by the Institutional Ethics Committee (RS1807/22(2783), RS1439/20(2434), RS203/IRE/24 PNRR, and RS N. 1777/22). Upon enrollment, each participant was assigned a unique identifier, and their samples and data were pseudoanonymized, processed and stored, and processed in accordance with relevant privacy and data protection regulations, including the European Union’s General Data Protection Regulation (GDPR), where applicable. Access to reidentifiable data was strictly limited to authorized research teams. Any subsequent use of biobank material required additional ethics approval and had to be consistent with the scope of the original informed consent.

### 2.3 Different biospecimen types collected at BBIRE

Tissue samples were obtained from surgical specimens. They include freshfrozen, formalinfixed, paraffinembedded (FFPE), frozen, and optimal cutting temperature (OCT) tissue. Freshfrozen tissue was obtained by sectioning tumor samples immediately after surgery (within 30 min), and when needed, the samples were immersed in RNAlater stabilization solution (Thermo Fisher Scientific, MA, United States) and incubated overnight at 4 °C to preserve RNA integrity. The samples were frozen in liquid nitrogen and then stored at 80 °C until further analysis (e.g., RNA/DNA extraction, proteomics). This method preserves molecular integrity, making it suitable for high quality nucleic acid and protein extracts. The 30 min limit was applied because the duration of cold ischemia is critical for preanalytical variables affecting the molecular integrity of samples (Berrino et al., 2020). For the tissue freezing methods, the samples were placed in cryotubes and frozen in liquid nitrogen without embedding medium. This approach preserves molecular integrity and is suitable for high quality nucleic acid and protein extracts. FFPE samples were fixed in 10% neutral buffered formalin (NBF) at 4 °C for 24 h after excision. After fixation, the samples were dehydrated with a graded ethanol series, cleared in xylene, and embedded in paraffin wax according to standard histopathological protocols. The FFPE blocks were stored at room temperature until sectioning. For the downstream analyses, 45 µm thick sections were cut with a rotary microtome and mounted on positively charged glass slides. The selected samples were embedded in optimal cutting temperature (OCT) compound (Kaltek, Italy), frozen in isopentane precooled with liquid nitrogen, and stored at 80 °C for optimal tissue morphology. They are suitable for cryosectioning and molecular analyses. In EC cases, tissue is harvested to ensure that the neoplastic cells are representative of the main tumor mass. Furthermore, a control sample of normal endometrial tissue was collected (at a specific distance from the tumor margin). Blood samples from hospitalized patients are collected by the nursing staff after they are requested by the physician through electronic blood analysis request forms. Blood samples are then sent to the Clinical Pathology Unit for centrifugation, aliquotation and storage by the BBIRE team. Detailed clinical and pathological records are linked to each sample to ensure reliable traceability. The BBIRE Biobank infrastructure guarantees high quality sample management and accessibility for approved research projects.

### 2.4 Establishment and maintenance of PDOs

PDOs were generated inhouse in the Translational Oncology Research Unit via optimized protocols adapted for EC tissue (not available from public repositories). PDO generation was achieved via the mechanical and enzymatic dissociation of fresh viable tumor cells. Specifically, single cell suspensions were obtained via a tumor dissociation kit (Miltenyi Biotec, Germany) according to the manufacturer’s instructions. The resulting cell suspension was then filtered with a 70 µm sieve (Miltenyi Biotec). The dissociated cell clusters were centrifuged at 1,200 rpm for 5 min, washed once with PBS and centrifuged again at 1,200 rpm for 5 min. If the pellet had a visible red color, erythrolysis with ammonium chloride solution (STEMCELL Technologies, Vancouver, Canada) was performed before the washing step. The dissociated cell clusters were resuspended in cold Matrigel (Corning Inc., Corning, NY, United States) and seeded in a prewarmed 24 well plate at a density of 6 × 105 cells per 30 µL droplet. The droplets were solidified for 30 min at 37 °C and 5% CO2 in the incubator, and then, 500 µL of organoid culture medium (Advanced DMEM/F12 (Gibco^TM^, Thermo Fisher Scientific, Waltham, MA, United States), 200 mM GlutaMAX (Gibco^TM^), 1 mM HEPES (Gibco^TM^), RSpondin 1 (HumanKine, Proteintech, IL, United States), 100 ng/mL Noggin (HumanKine), 1.25 mM Nacetylcysteine (Sigma‒Aldrich, Merck KGaA, Darmstadt, Germany), 5 mM nicotinamide (Sigma‒Aldrich), 1X B27 supplement (Invitrogen^TM^, Thermo Fisher Scientific, MA, United States), 1X N2 supplement (Invitrogen^TM^, Thermo Fisher Scientific), 1% chemically defined lipid concentrate (Gibco^TM^), 250 nM A8301 (HumanKine), 50 ng/mL EGF (HumanKine), 40 ng/mL IGF1 (HumanKine), 20 ng/mL HGF (HumanKine), 5 ng/mL IL6 (HumanKine), 100 nM SB202190 (Sigma Aldrich), 10 nM 17 βestradiol (Sigma Aldrich), and 10 mM Y27632 (HumanKine) were added to each well. PDOs were kept in a controlled incubator (37 °C, 5% CO_2_) and routinely monitored for morphology and viability. The PDOs were transferred every 5‒9 days depending on the proliferation rate. For molecular analyses, nucleic acids were extracted from freshfrozen and/or OCTembedded tissue as well as from PDOs via standardized Qiagen AllPrep DNA/RNA kits/miRNA universal kits (Qiagen, Germany) and the manufacturer’s protocols to ensure optimal purity and yield. We assessed DNA and RNA concentrations and quality via Qubit fluorometry (Invitrogen^TM^, Thermo Fisher Scientific) and evaluated the integrity of nucleic acids via capillary electrophoresis via the Agilent TapeStation system (Agilent Technologies, CA, United States). The extracted nucleic acids were stored at 80 °C until further use.

### 2.5 Immunohistochemical analysis

PDOs were resuspended in 10 mL of Cell Recovery Solution (Corning, Inc.) and incubated on ice for 1 h. The samples were subsequently centrifuged at 500 × g for 5 min at 4 °C. The supernatant was removed, and the pellet was resuspended in 10 mL of ThinPrep solution. Afterward, the samples were processed via the Cellient^TM^ Automated Cell Block System (Hologic, Inc., MA, United States), which is fully automated and creates a paraffinembedded cell block via isopropanol for dehydration and xylene for clarification. The cell blocks were cut into 3 μm sections via a Leica SM 2000R microtome (Advanced Research Systems Inc., PA, United States) and mounted onto slides. The slides were dewaxed in xylene, rehydrated through a series of graded ethanol solutions and stained with Gill’s hematoxylin (BioOptica Milano S.p.A., Italy) and eosin (BioOptica Milano S.p.A.). The slides were incubated with the following primary antibodies: estrogen receptor (clone 6F11, Leica Biosystems, Germany), progesterone receptor (clone 16, Leica Biosystems), TP53 (clone DO7, Leica Biosystems), vimentin (clone V9, Leica Biosystems), and CKAE1/AE3 (clone AE1/AE3, Leica Biosystems) in an automated immunostainer (BondIII, Leica Biosystems) according to the manufacturer’s instructions. Citrate buffer, pH 6 or pH 8, was used to unmask the antigens in each case. Images were obtained at a magnification of 20× by using the Aperio Image Scope system (Leica Biosystems) equipped with Digital Image Capture software (RRID:SCR_020993 scicrunch.org). The evaluation was based on the percentage of positive cells.

### 2.6 Digital pathology

For each input sample, the original diagnostic slide was produced from a 45 μm section cut from a formalinfixed, paraffinembedded wholeslide block and stained with H&E. The slides were then digitized at 0.25 μm/pixel (40×) via an Aperio AT2 scanner (Leica Biosystems) under default instrument settings: automatic autofocus, a single focal plane, and no Zstacking. Images were stored in the proprietary SVS (scanned virtual slide) format and uploaded to a local WSI (wholeslide image) management server (eSlideManager, Leica Biosystems). All the slides were scanned in pseudoanonymized form, and their analysis had no bearing on patient management. A pretrained model classifies WSI areas into three classes: tumor, stroma, and immune (Gallo et al., 2024).

### 2.7 Whole exome and transcriptomic sequencing analyses of tumor samples and PDOs

Nextgeneration sequencing (NGS) analyses, including whole exome sequencing (WES) and transcriptomic profiling, were performed on tumor tissue samples and PDOs. Our institute’s NGS facility provides expert support for advanced translational research projects. Genomic DNA and total RNA were extracted via validated protocols optimized to ensure high yield and purity. For WES, DNA libraries were prepared via the Twist Comprehensive Exome Kit according to the manufacturer’s instructions (Twist Bioscience, CA, United States). The libraries were sequenced on an Illumina NovaSeq 6000 (Illumina, CA, United States) platform to generate pairedend reads. RNA sequencing (RNAseq) libraries were prepared via the Illumina Stranded Total RNA Prep with RiboZero Plus kit (Illumina). These libraries were then sequenced on the Illumina platform to produce pairedend reads with a target depth of approximately 100 million (WES) and 50 million reads (RNAseq) per sample.

### 2.8 Bioinformatic analysis

The RNAseq and WES samples were processed with a fully containerized, version controlled nfcore workflow. Specifically, nfcore/rnaseq was used for transcriptomics, and nfcore/sarek was used for variant calling. Both pipelines were executed with NextFlow under Docker/Singularity containers pinned to exact image digests, guaranteeing complete provenance and bit reproducibility in line with the nfcore bestpractice guidelines (RRID:SCR_024135). Each run generated a comprehensive MultiQC, which was thoroughly inspected before proceeding with further analysis (RRID:SCR_014982). Specifically, residual rRNA must be <10%, the 5′:3′ coverage ratio must be ≈1.0, the number of PCR duplicates must be <30%, and ≥90% of the reads (with ≥70% proper pairs) must align to the reference. These cutoffs come directly from ENCODE best practice standards (Hitz et al., 2016). Libraries that miss any threshold are subjected to resequencing or discarded, ensuring that downstream analyses rely on high quality starting material.

## 3 Results

### 3.1 Overview of the BBIRE biobank and collaborative network

**FIGURE 2:**
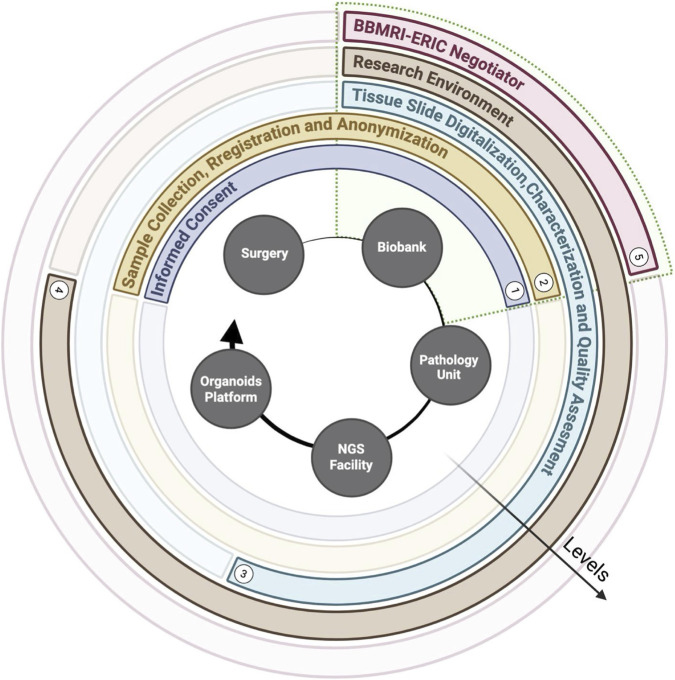
Circle plot of the biobanking workflow. The filled gray circles denote the operational units arranged in sequential order. Concentric levels 15 indicate the main workflow stages; each level spans the units involved in that stage.

The BBIRE biobank was established to enable systematic and high quality collection, processing and distribution of tumor samples. Since 2017, in close collaboration with the Department of Gynecologic Oncology, BBIRE has supported the prospective enrollment of samples from patients with gynecologic malignancies, including EC, for advanced molecular profiling in translational medicine. When specified by standard protocols, selected sample samples are transferred to the NGS facility and/or the Translational Oncology Research Unit, where they are processed for both molecular analyses and/or the generation of PDOs and other functional models, supporting downstream translational research and precision oncology applications. Figure 2 summarizes the workflow, with the main steps standardized for traceability. BBIRE is regulated by a quality manual and internal rules, as established by institutional resolutions No. 431 (13 June 2017), No. 697 (4 September 2018), and No. 1360 (30 December 2021). These documents designate two working groups within the biobank: the steering committee (SC) and the operational group (GRO). The SC supports the Scientific Directorate in evaluating and selecting research projects submitted by investigators seeking to utilize or provide human biological samples for research purposes. The Operational Group supervises the two primary areas of activity: tissue biobanking and liquid biobanking. This group coordinates daily operations, implements and maintains quality management and certification or accreditation processes, and defines SOPs and quality control measures for all collected samples. BBIRE is embedded within an interconnected and collaborative framework of international biobanking networks. Access to biospecimens within these networks is regulated through transparent and structured evaluation processes that align with international standards. Access to BBIRE samples follows a multistep evaluation process integrated within the BBMRI-ERIC Federated Platforms. After identifying samples through the BBMRI-ERIC platform, investigators submit their requests via the BBMRI-ERIC Negotiator. This tool enables direct interaction between applicants and the biobank, allowing both parties to clarify feasibility and eligibility criteria. Submitted projects undergo an initial administrative and technical assessment by the Biobank Operational Group, which verifies the completeness and consistency of the requested information. Then, requests are evaluated by the Steering Committee for scientific relevance and ethical compliance. Upon approval, the release of samples and data is regulated by formalizing institutional agreements, including Material Transfer Agreements (MTAs) and Data Transfer Agreements (DTAs). When multiple requests compete for limited biospecimens, the Steering Committee prioritizes projects based on scientific merit, methodological robustness, and sample availability.

The BBIRE workflow is a comprehensive, multilayered approach designed to ensure the ethical collection, preservation, and use of EC samples. It incorporates several essential components: the surgical suite, where specimens are collected; the biobank, where samples are stored and monitored; the pathology unit, which conducts histological and immunophenotype validation; the NGS facility, which generates high throughput molecular data; and the organoid platform, which uses patient derived models to mimic tumor biology. Concentric levels surrounding this core process ensure rigor and reproducibility. The process begins with informed consent, followed by sample collection, registration, and pseudoanonymization, to uphold ethical standards and maintain traceability (GDPR (Reg. UE 2016/679)) (EDPB | European Data Protection Board, n.d.). Each sample was characterized, and a quality assessment was performed to ensure that only well-defined materials were subjected to further analyses. Beyond the laboratory, the BBIRE workflow is part of a larger research ecosystem that encourages collaboration and standardization. This patient centered system ensures high quality biospecimens and fosters a trusting relationship between patients and researchers. BBIRE integrates surgery, pathology, biobanking, sequencing and organoid platforms within a unified ethical and technical framework to transform individual contributions into collective advances in precision oncology.

### 3.2 Standardized collection and processing of gynecologic cancer biospecimens at BBIRE

**FIGURE 3:**
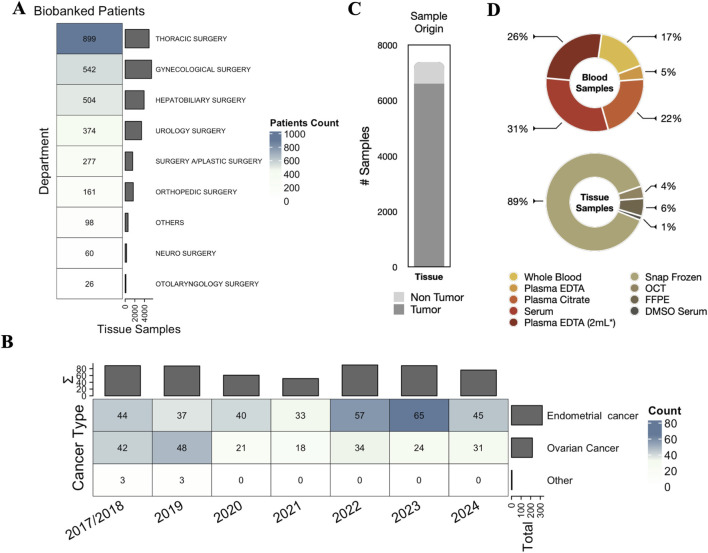
IFOIRE Biobank summary. **(A)** Patients stratified by pathology with annotated totals of tissue and serum samples. **(B)** Pie chart of the tissue sample origin. **(C)** Pie chart of preservation categories. **(D)** Annotated heatmap of gynecological samples across pathologies by year.

The BBIRE biobank stores a wide range of cancer samples, with a focus on gynecological malignancies. Figure 3A provides an overview of patient numbers by pathology and illustrates both the diversity of samples and the frequency of preservation methods used. Approximately 50% of stored samples are distributed based on collaboration requests from internal and external laboratories (DR60 Research Project Management). Figure 3B shows the annual distribution of gynecologic cancer cases from 20172024, highlighting fluctuations that may reflect changes in incidence or diagnostic practice. EC is the most representative malignancy, making the biobank a valuable resource for translational research. The continuous availability of EC samples has supported molecular and clinical studies, including research on genomics, treatment response and biomarker identification. A total of 545 patients with gynecological malignancies, including endometrial, ovarian, and vulvar cancers, were enrolled in the BBIRE biobanking program following ethical approval and disease specific informed consent. Most of the enrolled patients had EC (59%), followed by ovarian cancer (40%) and other gynecological cancers (<1%) (Figure 3B). Biobank groups, surgical teams, pathologists, and molecular and bioinformatics units must collaborate to standardize and enhance biospecimen handling. This collaboration transformed routine procedures into valuable resources, allowing the acquisition of well characterized tissue and blood samples from gynecological patients for future research. A total of 6,762 tissue samples from 542 patients with gynecological cancers were collected and stored under different conditions, such as cryopreservation and paraffin embedding. Among these samples, 3,348 were obtained from patients with EC and processed according to standardized protocols. Specifically, 2,854 samples were cryopreserved, 179 were embedded in optimal cutting temperature (OCT), 63 were preserved in dimethyl sulfoxide (DMSO) serum, and 252 were stored in FFPE tissue. Ultimately, 780 nonpathological EC tissues were preserved: 776 were cryopreserved, and four were embedded in optimal cutting temperature (OCT) compound (see Figures 3C,D). This thorough representation underscores the clinical importance and growing research focus on EC, establishing a robust foundation for molecular stratification, biomarker discovery, and personalized therapeutic approaches. Additionally, from 2017 to 2024, a total of 11,335 blood and derivative samples were collected, including whole blood (1,932), EDTA plasma (3,436), citrate plasma (2,458), and serum (3,509). The extensive range and depth of the collected materials position EC as a prime model for implementing precision oncology strategies within BBIRE. Standardized protocols were utilized for processing, aliquoting, and storing tissue and blood samples to ensure quality and reproducibility. Strong data management and security measures were put in place to guarantee accurate sample tracking and maintain patient confidentiality.

### 3.3 Verified biobank protocols empower preclinical model development

**FIGURE 4:**
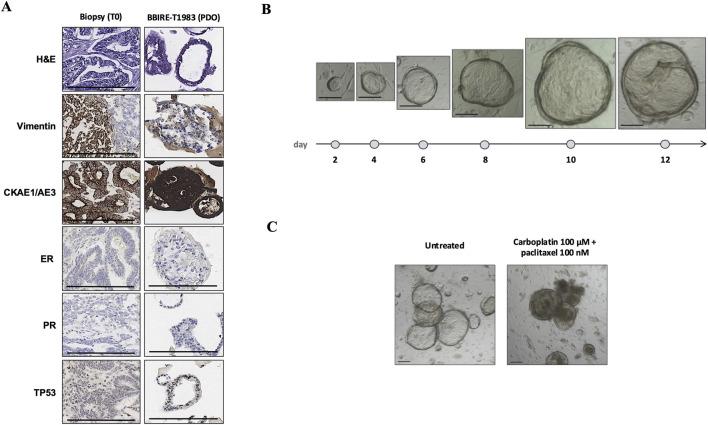
Characterization and drug response of PDO ECs. **(A)** IHC comparison between the original biopsy sample (T0) and the matched PDO sample BBIRET1983. Representative images showing H&E staining and immunostaining for Vimentin, CKAE1/AE3, ER, PR, and TP53. Scale bars = 100 µm. **(B)** Representative brightfield images illustrating PDO growth dynamics over 12 days in culture. Scale bars = 100 µm. **(C)** Representative images of PDOs after treatment with carboplatin (100 µM) plus paclitaxel (100 nM) compared with untreated controls, showing morphological alterations and reduced viability under the treated conditions. Scale bars = 100 µm.

**FIGURE 5:**
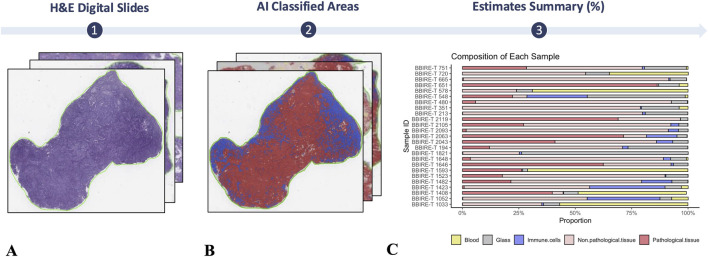
Digital pathology. **(A)** Representative digitized wholeslide image of an endometrial tissue section used for morphometric analysis (hematoxylin and eosin, H&E). **(B)** The section in **(A)** after image analysis, shown with a pseudocolor overlay highlighting regions classified as cancer, normal, or immune cells. **(C)** Percentage estimates for endometrial biobank samples.

The biobank’s operational performance was evaluated via defined verification parameters. The annual sample volume and traceability were consistently monitored to ensure accurate documentation and realtime tracking of all the samples. This enabled comprehensive traceability of samples from acquisition to storage and guaranteed compliance with quality and ethical standards. The cryopreservation of fresh, frozen tissue fragments is key to the long term storage of viable material and enables its use in translational medicine and research. Our data from 20172024 confirm the importance of optimizing cryopreservation protocols to maintain tissue viability, especially for applications such as PDO models. Tissue viability and subsequent propagation depend on cryopreservation conditions, particularly the freezing medium and cooling rate. The most successful results were obtained with a controlled rate freezing protocol set at 1 °C per minute down to −80 °C, in accordance with the SOPs. The success rates of these procedures range from 50% to 80%, allowing tissue viability to be preserved. With respect to sample traceability and annual recruitment, 91 patients (57 with EC and 34 with ovarian cancer) were recruited in 2022, for a total of 1,014 samples stored for downstream use and distribution. Morphologic and phenotypic validation was performed on 458 samples at T0 (time of sample receipt) within BBIRE, all of which showed concordance with the expected diagnostic features. A subset of 154 samples distributed externally were subjected to a similar check. These samples were found to be 100% matched for adenocarcinomas and EC cases, whereas 70% of melanomas and 60% of breast cancer samples matched the expected phenotypes. Importantly, EC tissue stored and analyzed at T1 also retained its complete morphological and phenotypic correspondence (Figure 4). To assess the preservation of tissue integrity and phenotype following biobanking, PDOs generated from a T1 sample were compared with the corresponding baseline tumor biopsy samples (T0) (Figure 4A). Histological and immunohistochemical analyses demonstrated that PDOs retained the principal morphological and molecular characteristics of the parental tumor. H&E staining confirmed similar tissue architecture, while IHC revealed consistent expression of the epithelial marker CKAE1/AE3, the stromal marker vimentin, hormone receptors (ER and PR), and TP53 in both tumor tissue and PDOs. These results demonstrate that PDOs accurately recapitulate the histomorphological and immunophenotypic features of their tissue of origin. In culture, the PDOs exhibited progressive growth and expansion over 12 days while maintaining structural integrity. Compared with no treatment, functional testing with carboplatin and paclitaxel resulted in a marked loss of structural organization, underscoring the translational potential of PDOs as a preclinical platform for drug sensitivity assays (Figure 4C). These results demonstrate that properly preserved, clinically processed biospecimens retain sufficient integrity to generate PDOs that accurately represent the original tumor characteristics. To further analyze the EC tissue samples, a digital pathology pipeline was used, following previously described methods (Gallo et al., 2024). This approach reduces variability and enhances reproducibility in tumor cell quantification for testing and immune infiltration assessment for prognosis (Figure 5).

### 3.4 Validated quality parameters ensure the robustness of multiomics data for translational use

**FIGURE 6:**
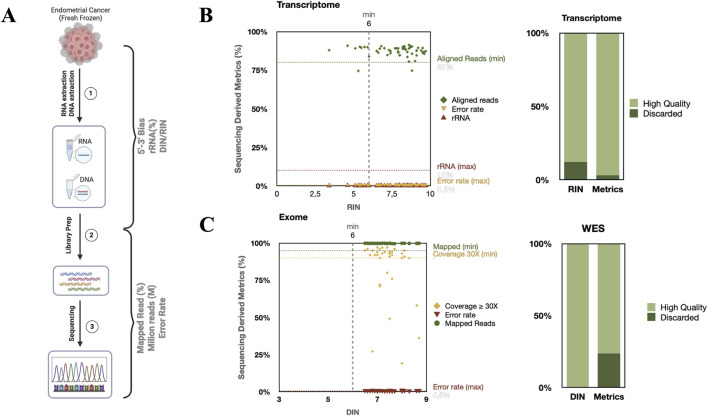
Quality assessment of biobank samples. **(A)** Metrics evaluated at each processing stage. **(B)** Transcriptome metrics vs. RIN with color-coded threshold lines; accompanying bar chart of dropout rates for the RIN and transcriptome metrics. **(C)** Exome metrics vs. DIN with color-coded threshold lines; accompanying bar chart of dropout rates for DIN and exome metrics.

We used strict, validated quality control steps to ensure that our transcriptomic and exome sequencing data were reliable and useful. As shown in Figure 6, we checked the quality at several points. For transcriptomics, we measured RNA integrity with the RNA integrity number (RIN) and DNA integrity with the DNA integrity number (DIN) via the Agilent TapeStation system. Following best practices, samples with a RIN or DIN ≥6 are usually suitable for further analysis. In our study, most RNA samples had a RIN ≥6, with a dropout rate of approximately 10%, confirming the high quality of the RNA and its suitability for reliable transcriptomic profiling (Figure 6B). DNA from matched samples also met integrity and concentration standards (dropout rate 0%), supporting its use in exome sequencing (see Figure 6C). Figure 6B is a scatter plot showing the relationship between RNA quality and sequencing performance. The quality cutoffs are marked with dashed lines. RIN >6, high fraction of aligned reads (>80%), minimal fraction of rRNA contamination (<10%) and low base calling error rate (<0.05%). Samples with an RIN ≥6 (indicated by a vertical dashed line) produced strong RNAseq results. The green diamonds represent the percentage of aligned reads. The orange inverted triangles represent the error rates, which remained below 0.5% in most cases. The red triangles represent rRNA contamination, which was also less than 10%. These results demonstrate that although an RIN/DIN ≥6 or higher typically predicts better outcomes, some samples just below this threshold also pass quality control (QC). The bar plot next to the scatter plot summarizes RNAseq performance by grouping samples as high quality (light green) or excluded (dark green) based on the RIN and sequencing results. Most samples that passed the RIN threshold also met all sequencing quality standards, demonstrating the effectiveness of our biobanking and processing procedures. Figure 6C shows how DIN affects sequencing coverage (>30X), mapped reads (>95%), and the error rate (<0.05%). Red inverted triangles indicate error rates, which were consistently low (<0.5%) in high integrity DNA. In contrast, degraded samples (DIN <6) presented reduced mapping efficiency, lower coverage, and higher error rates, negatively impacting downstream analyses such as variant calling. The rightmost bar plot illustrates the proportion of DNA samples classified as high quality (light green) or excluded (dark green). Most samples with DIN ≥6 were retained, whereas those with lower integrity were frequently discarded. Together, these data emphasize the critical role of nucleic acid integrity in achieving reliable, high quality sequencing outputs from EC tissues; however, they also highlight how some borderline samples (RINs 5 and 5) still reach optimal sequencing metrics. No direct correlation is found between DIN/RIN and these metrics.

### 3.5 Integrated data management and security measures

A comprehensive set of associated data is recorded and stored at the time of sample collection within the BBIRE Biobank. For each sample, owing to the complexity and large amount of information, a special software platform is required for standardized data acquisition, integration, and accessibility. The dataset includes demographic and clinical information (e.g., diagnosis, treatment history, and comorbidities); detailed metadata related to the samples (e.g., type of biological material, collection and processing method, storage conditions, and precise localization within the biobank); and additional annotations that increase the sample’s research value. Over time, this dataset has been enriched with molecular and phenotypic characteristics, such as DNA or RNA sequencing, cell line or organoid generation, and clinical followup updates. Data storage and management facilities must be open and easily accessible for upgrades. The installation of a biobank information management system (BIMS) that is strong, secure, and interoperable is pivotal in supporting the longitudinal integration and governance of these data. This digital infrastructure supports every stage of the biospecimen lifecycle, from consent and collection to analysis and sharing. It also helps meet GDPR requirements and other international biobanking standards. The BBIRE biobank uses a clear and organized process to manage access to biospecimens, following international biobanking standards. To access BBIRE samples, researchers go through several steps within the BBMRI-ERIC Federated Platforms. First, they find the samples they need using the BBMRI-ERIC platform and submit their requests through the BBMRI-ERIC Negotiator. This tool lets applicants and the biobank communicate directly to discuss feasibility and eligibility. The Biobank Operational Group then reviews each project to check that all information is complete and consistent. Next, the Steering Committee evaluates the requests for scientific value and ethical standards. If a project is approved, the release of samples and data is managed through formal agreements, such as Material Transfer Agreements (MTAs) and Data Transfer Agreements (DTAs). If there are multiple requests for the same limited samples, the Steering Committee gives priority to projects with strong scientific merit, solid methods, and available samples.

Once a sample is processed, a unique 2D barcode is assigned to it, allowing for automated tracking and registration within the biobank management software. Every movement, handling event, or update from this point on is recorded, facilitating the linking of new information with the corresponding biospecimen. Annotations in their entirety are carried out according to the rules of using standard terminologies, vocabularies, and biomedical ontologies for the purpose of ensuring both data consistency and the functionality of data interchange.

**FIGURE 7:**
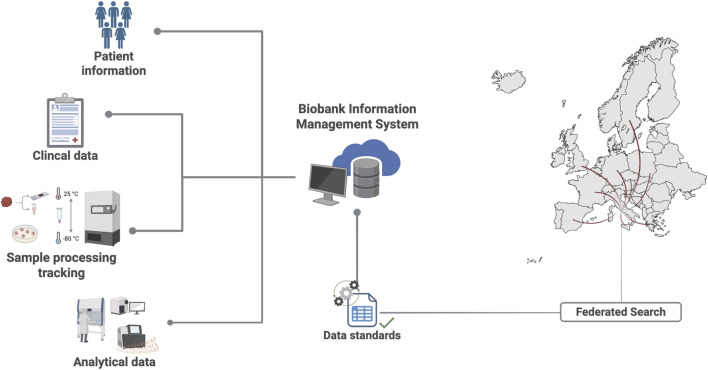
Biobank Information Management System (BIMS). Information flow and system interactions within the BIMS ecosystems.

Because the data stored in the biobank system are sensitive, security measures must be in place to protect and ensure the confidentiality of the data. These measures include role-based access control within the BIMS, user authentication, logging of all changes and processes related to samples and data, and identification of the user responsible for each action. With respect to data sharing and usage, methods are applied to minimize the necessary amount of information and to pseudonymize personal identifiers in accordance with ethical and legal standards. Access to data is granted only to authorized users under controlled conditions to safeguard participants’ privacy. The implementation of BIMS is therefore a key element in achieving a good digital maturity level within a biobank (Jacotot et al., 2022). Adopting such a system facilitates participation in national and international research networks and projects. By enabling efficient data retrieval and export, BIMS supports the implementation of ETL (extract, transform, load) processes, which are crucial for converting data into common data models (CDMs). These data formats are essential for interoperability because they ensure that data are standardized and accessible to both humans and machines. Moreover, these frameworks enable seamless integration with platforms such as the BBMRI Directory, which is a comprehensive catalog of biobank collections that exclusively displays information for users browsing (BBMRIERIC Directory/Locator RRID:SCR_004226) (BBMRIERIC). The BBMRI Locator is a federated tool for searching samples and associated data that adheres to the FAIR principle while remaining GDPR compliant. As shown in Figure 7, the architecture of the BIMS ensures seamless data flow and sample traceability via three modular components: (i) a frontend user interface for biobank operators and researchers, (ii) a secure relational database backend, and (iii) integration layers for interoperability with clinical and laboratory systems. This modular and scalable approach supports daily operations and allows the biobank to participate in multicenter studies and bigdata research initiatives.

## 4 Discussion

For many years, EC has occupied a marginal position in gynecological oncology and is often referred to as a “Cinderella” tumor. This characterization was primarily due to the contradictory features of the disease: although EC was the first invasive cancer of the female reproductive tract in the Western world, it has received very little research attention, is usually treated only surgically, and has very few options for systemic treatment in advanced stages. Moreover, the limitations of the histomorphology based classification system contributed even more to this underestimation, resulting in a lack of adequate clinical tools for risk stratification and treatment planning. The introduction of the TCGA (The Cancer Genome Atlas) molecular taxonomy has reversed this scenario and enabled the use of molecular features for better stratification of patient prognosis: the new FIGO classification 2024 accordingly changed the first steps of gynecological oncology toward personalized treatment (Colombo et al., 2024). In this evolving landscape, biobanks are essential infrastructures that link surgical practice, molecular profiling and translational research. The BBIRE biobank oversees the preanalytical standardization process and verifies the quality of the collected samples. This includes the validation of the molecular basket, the identification of new disease biomarkers and the testing of new therapeutic strategies. Moreover, biobanks are pioneering the integration of biological samples with imaging techniques and preclinical 3D models for the first time, as this is the only method for guaranteeing ethical compliance and multicenter collaboration. This is a priceless, high quality, and certified resource of knowledge that has enormous potential to accelerate EC research. They become exponentially more significant when they are integrated into international networks, as the collection of rare and diverse cohorts enables the rapid translation of molecular insights into precision tools. Therefore, it is feasible to direct research in EC, which has been the area of least great impact among gynecologic malignancies for an extended period, to finally garner the requisite scientific attention and innovation. In the field of EC, these aspects are especially critical, as the limited knowledge and multiomic background on how to predict individual risk necessitate the urgent ultra stratification of patients. Consequently, surgeons can identify the most effective surgical approach to preserve fertility in young patients, administer appropriate treatment and anticipate the emergence of drug resistance with the assistance of new insights (Adishesh et al., 2017; Adishesh and Hapangama, 2019; Zhu et al., 2021; Grech et al., 2025; Kitson et al., 2024; Betti et al., 2023; Bruno et al., 2023).

These advancements provide significant support for the development and testing of targeted therapies using patient derived preclinical models. In the era of artificial intelligence (AI), it has been argued that not only the quality of the data but also the quantity of data used to obtain accurate results. The advancement of precision oncology in EC will be contingent upon our ability to integrate AI applications with robust biobanking infrastructures, ensuring that the data are not only numerous but also standardized, reliable, and clinically relevant. In this view, biobanks are not merely repositories; they are hubs of innovation that (i) centralize research in referral centers and incorporate data from other low volume hospitals in a comprehensive and representative manner. Additionally, they (ii) improve quality by applying rigorous standards and expertise. The Institutional Tumor Molecular Bord (26) could serve as a valuable tool for the implementation of precision oncology in clinical practice, establishing an experimental clinical bridge between biobanks, preclinical models, and patient management. In a certified biobank setting, the standardization of EC sample collection procedures and management has become crucial/essential due to the potential significant impact on patient management and treatment. Although artificial intelligence and molecularly informed biobanking hold potential, several challenges must be acknowledged. First, the confidence for downstream analyses is compromised by inconsistencies in preanalytical procedures, including cold ischemia time, fixation, and storage conditions. Optimizing these preanalytical variables could indeed substantially increase the percentage of samples fit for purpose after biobanking. Second, a significant number of existing EC cohorts are small, sometimes highly selected, and not truly representative for training and validating the generalizability of AI-based models, particularly for underrepresented patient groups such as adolescents and young adults. Establishing networks across multiple infrastructures could partially address this limitation by expanding the pool of diverse and rare cases. Third, the simultaneous integration of multiomics layers with clinical and imaging data is challenging from a technical and computational viewpoint, emphasizing the urgent need for standardizing data harmonization techniques. In addition to these technical difficulties, ethical and legal restrictions, including data stewardship under the GDPR and consent requirements for broad data reuse, still hinder crossborder collaboration. Sustainability remains an ongoing challenge for biobanks. Maintaining high quality infrastructures, digital pathology, and multiomics pipelines requires sustainable long-term funding that is seldom guaranteed. Taken together, these challenges demonstrate that the path to precision oncology in ECs is not facilitated by algorithms alone but rather by the quality of the infrastructures that support them. By investing in standardized biobanking, building international networks, and ensuring sustainable stewardship, the field could transform EC from a long-neglected tumor to a flagship for data driven, patient centered oncology. In conclusion, a biobank model organized as BBIRE could truly represent the lost slipper in Gynecological Oncology’s “Cinderella Story” if it is placed in the appropriate hands to be restored and interpreted.

## 5 Conclusion

Biobanking is acknowledged as one of the major and leading tools that contributes to the advancement of cancer studies. Indeed, the fundamental function of the BBIRE/biobank is to provide scientists with high quality biospecimens, as these samples serve as the foundation for the molecular analysis of cancer cells. The future of molecular and translational research hinges on the supply of these samples, which are complemented by clinical data and patient outcomes in everyday situations. This combination makes it possible for researchers to locate new biomarkers, produce targeted therapies and personalized treatment plans, improve patient care and advance effective cancer treatment. Oftentimes, scientists working on research projects need to share samples (which can be biological specimens, datasets, or materials) with each other. Proper sample distribution and sample sharing are successful for all collaborators, who can thus obtain the material under optimal conditions in a timely manner. Accessibility, in this context, means ensuring that these samples are readily available and are easy for the authorized team members to use, which in turn facilitates the smooth running of the work.

## Data Availability

The data presented in the study are deposited in the ENA repository, accession number PRJEB107727 (secondary accession ERP188619).
